# Storage Stability of Conventional and High Internal Phase Emulsions Stabilized Solely by Chickpea Aquafaba

**DOI:** 10.3390/foods11111588

**Published:** 2022-05-28

**Authors:** Graziele Grossi Bovi Karatay, Andrêssa Maria Medeiros Theóphilo Galvão, Miriam Dupas Hubinger

**Affiliations:** Department of Food Engineering and Technology, School of Food Engineering, University of Campinas (UNICAMP), Monteiro Lobato Street, 80, Campinas 13083-862, Brazil; a264692@dac.unicamp.br (A.M.M.T.G.); mhub@unicamp.br (M.D.H.)

**Keywords:** pulses, emulsifier, stabilizers, oil structuring, HIPE, aquafaba

## Abstract

Aquafaba is a liquid residue of cooked pulses, which is generally discarded as waste. However, it is rich in proteins and, thus, can be used as a plant-based emulsifier to structure vegetable oil. This study investigates chickpea aquafaba (CA) as an agent to structure different oil phase volumes (Φ) of canola oil (CO). CO was structured in the form of conventional emulsions (EΦ65% and EΦ70%) and high internal phase emulsion (HIPE) (EΦ75%) by the one-pot homogenization method. Emulsions were evaluated for a period of 60 days at 25 °C in terms of average droplet size (11.0–15.9 µm), microscopy, rheological properties, and oil loss (<1.5%). All systems presented predominantly elastic behavior and high resistance to coalescence. EΦ75% was the most stable system throughout the 60 days of storage. This study developed an inexpensive and easy to prepare potential substitute for saturated and trans-fat in food products. Moreover, it showed a valuable utilization of an often-wasted by-product and its conversion into a food ingredient.

## 1. Introduction

Cooking pulse seeds in water, canning, as well as hummus production, yields an inexpensive, viscous, rich liquid called aquafaba [1,2]. Aquafaba is an eco-friendly by-product, rich in nutrients, with vast potential to be used as a food ingredient due to its emulsibility, foamability, gelation, and thickening properties [2]. Aquafaba’s properties are attributed to its composition consisting of protein, polysaccharide, polysaccharide-protein complexes, coacervates, saponins, and phenolic compounds [1,3,4,5]. This composition comes from the components transferred from seed to water during cooking, as well as from the interactions between these components under high pressure and temperature [6,7,8]. Moreover, this composition is greatly influenced by pulse cultivar and cooking and soaking conditions [6]. 

Using aquafaba as a food ingredient is a “win–win–win–win” situation as (i) it is environmentally sustainable since it enables the utilization of a by-product that is generally discarded by end consumers; (ii) it is nutrient rich; (iii) it is a cheaper source of protein when compared to animal-based- and plant-based-protein concentrates, isolates, and hydrolysate; and (iv) it is suitable for vegan products, for which the market is expected to rapidly expand in the upcoming years. Additionally, the use of aquafaba is in line with the trend of green and clean labeling, and it is promising for a broad acceptance from a consumer awareness point of view. 

The emulsifying activity, capacity, and stability of chickpea aquafaba (CA) has been reported in various studies [5,9,10]. He et al. [6] evaluated different chickpea cultivars to assess its influence on the emulsification properties of CA-based emulsions with an oil phase volume (Φ) of 70%. The authors reported that the emulsion capacity and stability among the five chickpea cultivars ranged from 1.10 to 1.30 m^2^/g and 71.9 to 77.1%, respectively. Lagarfa et al. [11] evaluated the influence of pH (3.5, 5, and 6.5) and chickpea-to-water ratio (1:1.5 to 1:5.0) during cooking on the emulsifying capacity of CA-based emulsions (Φ = 60%). The authors reported values within the range of 3.9–72.3% for emulsion capacity and 0.0–76.3% for emulsion stability, whereas Huang et al. [12] reported a 46% emulsifying activity of CA-based emulsions (Φ = 50%). The results from these different studies show the important role pH, chickpea/water ratio, Φ, and cultivars play on the emulsifying properties of CA-based emulsions. 

Even though there have been reports on CA emulsifying properties, emulsion properties have only been partly elucidated and there is no in-depth study evaluating the stability of CA-based structured oils produced with different oil phase volumes over longer periods of storage. It is well known that one of the most difficult problems to solve in emulsification technology is the creation of stable emulsion structures that can resist prolonged storage without breakdown through physical instability mechanisms, such as gravitational separation and droplet aggregation [13]. Therefore, it is necessary to study such systems for longer storage periods in terms of its physicochemical and rheological behaviors to adequately evaluate its potential as a fat replacer.

Among the different emulsion types, high internal phase emulsions (HIPEs) have attracted considerable interest to be used in food applications due to their elevated fat/water content (minimal Φ of 74%) and adjustable viscoelasticity [13,14]. One important application of HIPEs is as an alternative to partially hydrogenated oils (PHOs), and, in turn, trans-fatty acids (TFAs) as hydrogenation leads to the production of TFAs [13]. Recently, protein-stabilized HIPEs have been developed using a simple one-pot homogenization method, in which mixtures of oil and aqueous protein solution are sheared for short times [14,15,16]. In this context, this study aims to evaluate the use of chickpea aquafaba (CA) from a Brazilian cultivar (BRS Aleppo) as a structuring agent to structure canola oil (CO) in the form of conventional emulsions and HIPE through the one-pot homogenization method and its storage stability for a period of 60 days at 25 °C. 

## 2. Materials and Methods

### 2.1. Materials

A kabuli type chickpea cultivar (*Cicer arietinum* (L.), var. BRS Aleppo) was kindly donated by Embrapa Vegetables (Brasilia, Brazil). All the seeds were frozen until the analyses. Canola oil (CO) (purity 100%; Seara Alimentos S.A, Sao Paulo, Brazil) was bought in a local supermarket (Dalben, Campinas, Brazil). Nile red, fluorescein isothiocyanate (FITC) and sodium azide were obtained from Sigma-Aldrich (St. Louis, MO, USA) and all other chemical reagents were of analytical grade. 

### 2.2. Chickpea Aquafaba (CA) Production

First, the chickpea seed was manually cleaned to remove broken seed, dust, and other foreign materials. Then, 400 g of seed was presoaked in distilled water at a chickpea:water ratio of 1:3 (*w*/*w*) for 16 h at 5 °C. Subsequently, 400 g of presoaked seed was rinsed with distilled water and mixed with distilled water at a chickpea:water ratio of 1:2 and cooked in a pressure cooker (Instant Pot^®^ 7 in 1 multi-use programmable pressure cooker, IPDUO60 V2, 6 quart/liters) at 115–118 °C (an autogenic pressure range of 70–80 kPa) for 30 min. These conditions were chosen based on a previously reported study that evaluated the optimum conditions for producing CA with the best emulsion quality [1]. Following cooking, the cooked chickpeas were kept in the cooking pot for 6 h. The CA was then drained using a strainer and separated from the cooked chickpeas, weighed, and stored frozen until use.

### 2.3. CA Proximate Composition

The moisture content of the CA was measured in an infrared moisture analyzer (model MOC63u, Shimadzu, Japan) at 105 °C, until constant weight was reached [17]. For the determination of protein, ash, fiber, and fat, lyophilized CA was used, and the results were then converted to a wet basis. The total protein content of freeze-dried CA was quantified according to the Kjeldahl method using 6.25 factor for the conversion of nitrogen to protein. For ash determination, the samples were placed in previously weighed porcelain crucibles. The samples were then carbonized over a Bunsen burner and placed in a muffle furnace heated to 550 °C and left at this temperature for 6 h, and then transferred to a desiccator containing silica gel. After reaching room temperature, the crucibles containing the samples were weighed to determine the ash content by mass difference according to AOAC Official Method 923.03. For crude fiber determination, a modified Weende procedure was used. In short, the samples were first boiled in sulfuric acid (1.25%) for the extraction of sugar and starch (acid digestion). The samples were then filtered and washed with water to remove acid residues and neutralize the pH. Subsequently, the samples were boiled with 1.25% sodium hydroxide to remove proteins, hemicellulose, and lignin (alkali digestion). The samples were again filtered and washed with water to remove alkali residues and neutralize the pH. The samples and filter were then dried at 100 °C and then at 550 °C in a muffle furnace. Crude fiber was then determined by mass difference (AOAC Official method 930.10). The extraction of the lipid fraction was performed in accordance with the methodology described by Bligh and Dyer [18]. The total carbohydrate content was calculated as the difference between 100 and the sum of the percentage of moisture, ash, lipid, and protein. All chemical analyses were performed in three replications.

### 2.4. Conventional Emulsions and HIPE Production

Conventional emulsions and HIPE formulated with CO and CA containing a concentration of 0.05% (*w*/*w*) of sodium azide (for the inhibition of growth of microorganisms) were prepared by a one-pot homogenization method using a rotor-stator device (Ultraturrax^®^ T18 basic, IKA^®^-Werke GmbH & Co., KG, Staufen, Germany) operating at 15.500 rpm for 1 min. CO was structured in the form of simple emulsions, namely, EΦ65% (35%CA and 65%CO) and EΦ70% (30%CA and 70%CO), and high internal phase emulsion (HIPE), EΦ75% (25%CA and 75%CO). The emulsions and HIPE were produced in triplicate, stored at 25 °C, and characterized on selected days until 60 days of storage were reached.

### 2.5. Conventional Emulsions and HIPE Characterization

#### 2.5.1. Droplet Size

The dimensions of the droplets were determined by static light scattering using Mastersizer 2000 (Malvern Instruments Limited, Worcestershire, UK). The samples were dispersed in water with a refraction index of 1.33 and a rotation velocity of 2100 rpm at room temperature (25 °C). The equipment possesses a stand-alone computer that runs the Malvern software. The Malvern software controls the optical bench and dispersion units and analyzes the raw data from the optical bench to determine the size of the particles, which are presented in many different formats. In this study, the droplet size was reported in terms of size distribution, volume-weighted (D_[4,3]_) and surface-weighted (D_[3,2]_) diameters, and span, according to Equations (1)–(3), respectively:

(1) $$D_{\left[4,3\right]}=\frac{\sum n_{i}d_{i}^{4}}{\sum n_{i}d_{i}^{3}}$$

(2) $$D_{\left[4,3\right]}=\frac{\sum n_{i}d_{i}^{3}}{\sum n_{i}d_{i}^{2}}$$

(3) $$\mathrm{Span}=\frac{\left(d_{\left(90\right)}-d_{\left(10\right)}\right)}{d_{\left(50\right)}}$$

where *d_i_* is the droplet diameter, *n* the number of drops, and *d*_(10)_, *d*_(50)_, and *d*_(90)_ are the diameters at 10%, 50%, and 90% of cumulative volume.

#### 2.5.2. Optical and Fluorescence Microscopy

All the samples were imaged via optical and fluorescence microscopy using an optical microscope (Carl Zeiss, Axio Scopo A1, Aalen, Germany) at room temperature (25 °C). The images were examined in the software AxioVision Rel. 4.8 (Carl Zeis, Aalen, Germany). For fluorescence analysis, the samples were stained with 10 µL of Nile red (0.1 g/L in polyethylene glycol) and 10 µL of FITC (0.02 g/mL in ethanol). The protein was dyed with FITC (green) and CO with Nile red.

#### 2.5.3. Rheological Measurements

The rheological measurements of the samples were determined on an AR 1500 ex (TA Instruments, New Castle, PA, USA) using a 2° stainless-steel cone and plate (40 mm diameter and 47 µm gap). 

Apparent viscosity data as a function of shear rate were acquired by performing flow curves with shear rate values ranging from 0 to 300 s^−1^, with three sequential ramps: up–down–up cycles, respectively, aiming at the elimination of thixotropy. Data from the third flow curve were adjusted according to the power law model, according to Equation (4):

(4) $$\sigma =k\times \gamma^{n}$$

where σ is the shear stress (Pa); k is the flow consistency index (Pa·s^n^); *γ* is the shear rate (1/s), and *n* is the flow behavior index (dimensionless). All the measurements were performed in triplicate at 25 °C on day 0 (fresh) and after 3, 7, 14, 30, 45, and 60 days of storage.

The viscoelastic behavior of the emulsions and HIPE was investigated by small amplitude oscillatory measurements. First, a stress sweep was performed by logarithmically increasing the stress from 0.01 to 100 Pa at a frequency of 1 Hz to identify the linear viscoelastic region (LVR) of the samples. Further rheological parameters at the LVR, such as the storage (G′_LVR_) and loss (G″_LVR_) modulus, the limiting value of oscillatory stress (OSL), the loss-tangent (tanδ_LVR_), and the flow-point oscillatory stress (FPOS) and flow-point G (FPG) for all samples, were determined from the amplitude sweep measurements.

Frequency sweeps of 0.01–10 Hz were subsequently performed at 25 °C and a fixed strain value of 0.1 Pa (within the LVR). Data from the frequency sweep were adjusted according to a power law model according to Equation (5):

(5) $$G^{\prime }=k^{\prime }\times \omega^{n\prime }$$

where G′ (Pa) is the storage modulus, k′ (Pa·s^n^) is a constant, ω (rad/s) is the oscillation frequency, and *n*′ (dimensionless) is the slope in a log–log plot of G′ versus ω. Both frequency and amplitude measurements were performed in triplicate at 25 °C on day 0 (fresh) and after 3, 7, 14, 30, 45, and 60 days of storage.

Temperature sweep tests were performed in the range of 10 to 80 °C with a fixed strain value within the LVR and frequency of 1 Hz. All the measurements were performed in duplicate on day 0 (fresh) and after 3, 7, 14, 30, 45, and 60 days of storage. 

#### 2.5.4. Conventional Emulsions and HIPE Stability

The stability of the conventional emulsions and HIPE was quantified in terms of centrifugal oil loss. Approximately 1 g of sample was put into Eppendorfs, centrifuged at 8600× *g* for 30 min at 5 °C. Following centrifugation, free oil was removed and the sample mass without free oil was recorded and determined according to Equation (6):

(6) $$\mathrm{Centrifugal}\;\mathrm{oil}\;\mathrm{loss}\;\left(\%\right)=\frac{m_{i}-m_{f}}{m_{i}}\times 100$$

where *m_i_* is the initial mass of the sample and *m_f_* is the final mass of the sample without free oil.

### 2.6. Statistical Analyses

The experimental data were depicted as the means ± standard deviation and analyzed applying one factor analysis of variance (ANOVA) using Statistica 8.0 software (Stat Soft. Inc., Tulsa, OK, USA). Significant differences (*p* < 0.05) between means were detected using the Tukey test. Graphs were obtained with Microsoft Excel Office 2016.

## 3. Results and Discussion

### 3.1. CA Proximate Composition

Chickpea hulls work as a membrane that control mass transfer during the soaking and cooking processes. When these hulls are damaged, the release of chickpea seed components (e.g., protein and carbohydrate) into the water is facilitated [6,19]. In turn, the components released from the chickpea seed into the cooking water affects the CA composition and, consequently, the functional properties of the resulting CA. Therefore, determining the proximate composition of CA is of high importance.

The moisture composition of CA in this study was 94.38% ± 0.19 and is in accordance with other studies. Shim et al. [20] reported on the moisture content of 10 commercial canned chickpea products; the values were between 92.98% and 95.12%. He et al. [6] produced CA from 5 different cultivars under similar conditions to the present study, and the reported values were in the range of 92.4% to 94.2%. As for Raikos et al. [21], who reported on canned CA, the reported value was 94.97%. 

The protein content of CA in this study was 1.21% ± 0.04. Stantiall et al. [4] produced CA by soaking chickpea seed (CS) in a 1:3.3 weight ratio (CS:water) for 16 h and cooking in a 1:1.75 weight ratio (CS:water) for 90 min; they reported a protein content of 0.95%. In the studies of Mustafa et al. [22] and Raikos et al. [21], the protein content value reported was 1.5% and 1.3%, respectively. Bulh et al. [10], who reported on the CA composition declared by the producer (Salling Group, Brabrand, Denmark), reported a relatively high protein composition of 6.3%. 

As for the ash content, the determined value in this study was 0.49% ± 0.01, which agrees with the previously reported values in the literature, which were 0.4%, 0.5%, and 0.6% in [4,21,22], respectively. The fiber content of CA was reported in very few studies and was 0.69 ± 0.03 and 4.04 ± 0.09 in [21,23], respectively. In this study, the determined value was 0.51 ± 0.16.

The carbohydrate content in this study was 3.39 %. Mustafa et al. [22] reported a 4% CA composition for simple and complex carbohydrates; whereas, in [10], the composition of carbohydrates was considerably high (i.e., 15%). 

As for the fat content, in some studies, it was not detected or was below the detection limit [4,22]. However, in our study, fat content was detected and was 0.14 ± 0.01, which is in accordance with [23], who reported a value of 0.13 ± 0.03 and with [21], who reported a value of <0.1%. A study on boiled chickpeas [24] reported a significant loss of small fractions of fats upon boiling. This fat loss could have undergone two processes: (i) leaching out into the cooking water, such as the case in the present study; or, (ii) the fat was degraded during processing [4]. On the other hand, in the study of [10], a high fat content of 2.2 % was reported.

In summary, these differences in the proximate composition of CA are due to many factors, such as the chickpea cultivar and especially the processing conditions used to produce the aquafaba. These different compounds can be tailored to have unique functional properties according to the desired use of the AQ. In this study, this specific composition was evaluated for its ability to structure liquid oil.

### 3.2. Droplet Size and Optical Microscopy 

**Figure 1:**
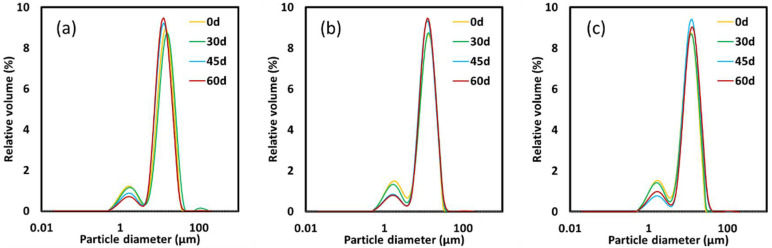
Droplet-size distribution of (**a**) EΦ65%, (**b**) EΦ70%, and (**c**) EΦ75% during 60 days of storage at 25 °C.

The droplet-size distribution for all formulations remained with a bimodal profile during the 60 days of storage (Figure 1). As can be observed, there were many overlapping curves throughout storage, indicating that the emulsions resisted prolonged storage without breakdown through physical instability mechanisms. 

Table 1 shows the droplet size, expressed in terms of D_[4,3]_ (based on the volume of a sphere) and D_[3,2]_ (based on the diameter of a sphere). The results of the droplet size in the present study were within the ranges of 11.0–12.8 µm, 12.3–13.0 µm, and 14.1–15.9 µm (D_[4,3]_) and 6.5–7.0 µm; 5.7–6.0 µm, and 5.4–5.7 µm (D_[3,2]_) for EΦ75%, EΦ70%, and EΦ65%, respectively. Although the average droplet sizes showed a significant difference (*p* < 0.05) as per the Tukey’s test, the variation in sizes between days 0 and 60 did not exceed 1.8, 0.7, and 1.8 µm (D_[4,3]_) and 0.7, 0.3, and 0.3 µm (D_[3,2]_) for EΦ75%, EΦ70%, and EΦ65%, respectively, which can be considered quite low. 

As compared to other studies that produced protein and protein-polysaccharide-based HIPEs using the one-pot homogenization method (under slightly different time and speed operating conditions), the results of this study produced emulsions and HIPEs with relatively smaller or comparable droplet sizes. For instance, Zuo et al. [16] produced HIPEs using sonicated quinoa protein isolate (QPI) and peanut oil (Φ = 80%) and reported D_[4,3]_ values within the range of 30–52 μm for HIPEs fabricated by sonicated QPI at low pH (3 and 5) and of 14–24 μm for those fabricated in neutral and alkaline conditions, which was the case for the present study in which the pH of aquafaba was quantified close to neutral (6.38 ± 0.01). In the study of Vélez-Erazo et al. [15], who produced sunflower oil (Φ = 74–96%) HIPEs with pea protein isolate and different polysaccharides (i.e., xanthan gum, carrageenan, gum Arabic, alginate, gellan gum, tara gum, locust bean gum, and pectin), D_[4,3]_ was in the range of 16.24–25.79 µm (depending on the polysaccharide used). These results show that, apart from being stable in terms of droplet size and distribution for 60 days of storage, emulsions and HIPEs produced in this study had considerably lower droplet sizes compared to other studies, which, in turn, can indicate higher stability during prolonged storage times. As for the Span values (measures of absolute width), all formulations presented a value of 1.6 throughout all the storage days, except for EΦ65% at day 30 and EΦ75% at day 60, for which the span value was of 1.7. 

**Figure 2:**
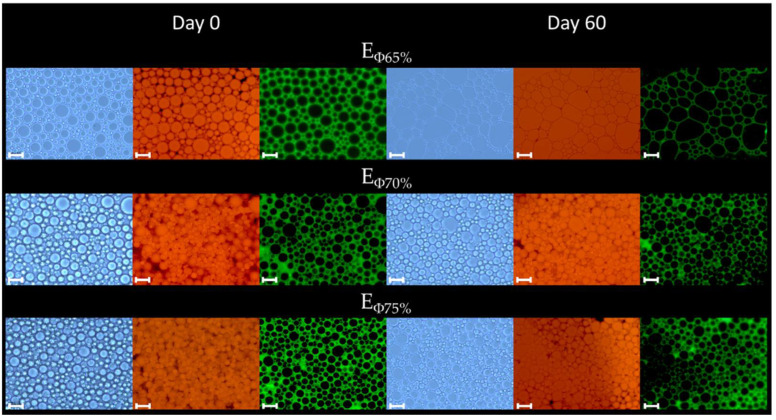
Optical and fluorescence microscopic images of E_Φ65%_, E_Φ70%_, and E_Φ75%_ at days 0 and 60; of storage at 25 °C (bar scale: 20 μm). Protein was dyed with FITC (green) and CO with Nile red.

A main difference between conventional emulsions and HIPEs is that the latter is a concentrated version of conventional emulsions, and typically has an internal phase (oil or water) volume fraction exceeding 74%, which is the case for the EΦ75% formulation in this study. At such a high concentration of oil, the oil droplets are tightly packed together and may adopt non-spherical shapes [13]. Nonetheless, in this study, the oil droplets in EΦ75% were mainly spherically shaped and did not present considerable differences from the conventional emulsions, EΦ65% and EΦ70%, which can be confirmed by optical microscopy (Figure 2). It can be observed that the oil droplets of EΦ65% increased considerably throughout the 60 days of storage. This was not evident in the droplet-size results (Table 1), but could be noted in the size distribution graph (Figure 1), in which the first peak (lower droplet sizes) decreased in terms of relative volume (%) and the second peak (higher droplet sizes) increased at longer storage days (i.e., days 45 and 60).

Moreover, it is possible to observe from the samples stained with FITC that the oil droplets were surrounded and coated with protein contained in the chickpea aquafaba (CA). It is likely that both the protein and the starch, as well as all other bioactive molecules, leached out from the chickpea seed into the cooking water during the cooking process were responsible for enabling emulsification and providing stability to the system, thereby preventing coalescence. 

It has been frequently reported that HIPEs with small particle sizes possess relatively high stability; therefore this parameter is suitable and has been used as an index to evaluate HIPE stability [25]. By taking this into account, it can be assumed that, as EΦ75% presented the smallest droplet size (confirmed by D_[4,3]_, D_[3,2]_ and the optical micrographs), it can be considered as the most stable system, followed by EΦ70% and EΦ65% in terms of size parameters. However, to further investigate the stability of these emulsions and HIPE, a combination of other parameters should be investigated.

### 3.3. Rheological Properties

#### 3.3.1. Steady-Shear Behavior

The flow curve parameters (shear stress and shear rate) were fitted to the power law model, and these are presented in Table 2.

With respect to the variation in the modeled parameters (i.e., k and *n*) within the 60 days of storage, there were significant differences (*p* < 0.05) among all the samples. The flow behavior index (*n*) values, which indicate the degree of non-Newtonian characteristics of the fluid, were between 0.31 and 0.50 (*n* < 1) for all the samples throughout storage, corresponding to a pseudoplastic behavior. At a higher oil concentration, the *n* values were lower, suggesting that the HIPE (EΦ75%) formed a more structured gel. The flow consistency index (k) is related to the apparent viscosity of the samples; the more viscous the sample, the higher the k. From our results, the k values were higher in the samples that contained more oil, which means that the HIPE (EΦ75%) was more structured and firmer than the emulsions (EΦ70% and EΦ65%), which can be confirmed by the apparent viscosity results at the shear rates of 5 and 300 per second (Table 2). These results can be related to the droplet size of the samples, as the bigger the droplet size, the lower the surface area available for droplets to interact, which, in turn, leads to lower shear thinning behavior and, consequently, lower viscosity [26,27].

Viscosity is an important parameter to evaluate, as it describes the flow properties of a system and can directly affect the appearance and the consistency of a product. It is directly dependent on strain rate; therefore, it is determined in terms of apparent viscosity and was expressed in this study at the shear rates of 300 s^−1^ and 5 s^−1^ (Table 2). Both emulsions and the HIPE showed shear-thinning behavior, which is characteristic of non-Newtonian fluids whose viscosity decreases under increasing shear rates. Moreover, at higher oil concentrations (EΦ75% > EΦ70% > EΦ65%) the shear stress was higher, resulting in increased viscosity, a parameter that can be a valuable feature depending on the desired application. With respect to the variation in the apparent viscosity both at the shear rates of 300 s^−1^ and 5 s^−1^ within the 60 days of storage, there was a significant difference (*p* < 0.05) for all samples except for EΦ75% (at a shear rate of 5 s^−1^).

**Figure 3:**
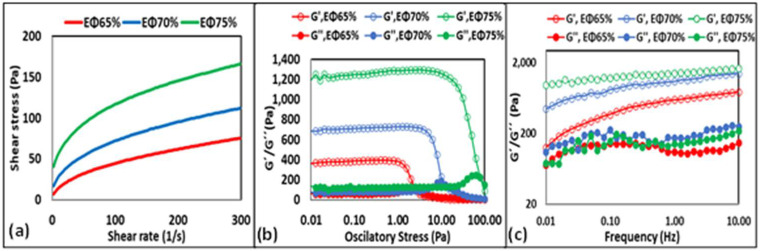
Flow curves plotted as shear stress versus shear rate (**a**), stress sweeps (**b**), and frequency (**c**) tests of EΦ65%, EΦ70%, and EΦ75% at day 0 of storage at 25 °C.

All the samples presented an initial shear stress (σ_0_) throughout storage (Table 2). With respect to the variation in the initial shear stress within 60 days of storage, there was a significant difference (*p* < 0.05) for all samples except for EΦ65%. The presence of σ_0_ indicates that the system needs an initial force to be applied for the sample to begin flowing. In turn, σ_0_ values are directly related to sample fluidity. Fluidity is defined as the ability of the sample to retain its appearance and structural integrity when inverted. In that sense, the higher the σ_0_, the higher the ability of the system to retain its appearance, which was confirmed by photographs taken directly after the samples were inverted (Table 2). As can be observed, both EΦ70% and EΦ75% presented higher σ_0_ and consequently lower mobility, when compared to EΦ65% at day 0 (Figure 3a) as well as throughout all the storage days. Higher fluidity indicates that molecules are less viscous and packed together to a lesser extent, which can be confirmed by the apparent viscosity results (Table 2) and the optical and fluorescence microscopic images (Figure 2). In summary, as EΦ75% is more viscous, and presented higher k values and lower *n* values, it can be considered as the most stable system, followed by EΦ70% and EΦ65%.

#### 3.3.2. Oscillatory Shear Behavior

##### Amplitude Sweep

An amplitude sweep test was conducted by varying the oscillatory stress (0.01–100 Pa) at a fixed frequency (1 Hz). This determined the linear viscoelastic region (LVR), in which G′ (storage modulus) and G″ (viscous modulus) were almost constant, and the nonlinear region, in which G′ and G″ started to decrease [28]. The oscillatory stress value, at which G′ sharply decreased, is defined as the critical oscillatory stress, also known as the limiting value of oscillatory stress (OS_L_). On day 0 (Figure 3b), at low-stress values (0.01–1 Pa), the G′ value of EΦ75% was the highest (~1250 Pa), showing higher elastic behavior when compared to EΦ70% and EΦ65%, which presented G′ of ~700 Pa and 380 Pa, respectively. When the stress was increased (>1 Pa), it was possible to identify the OS_L_ values, which were 1, 5, and 20 for EΦ65%, EΦ70%, and EΦ75%, respectively. Determining the OS_L_ value is important as it determines the maximum deformation that a system can withstand without structural breakdown [28]. Results of this study show that EΦ65% (lower OS_L_) is the first to lose structure, followed by EΦ70% and EΦ75%. This outcome indicates that an increase in the oil concentration improves the strength and rigidity of the system.

To evaluate if the samples maintained their initial structure throughout the 60 days of storage, further rheological parameters at the LVR were determined from the amplitude sweep measurements (Table 3).

The results show that, for all three formulations, the OS_L_ decreases considerably, indicating that samples lose their structure at lower oscillatory stress levels. The values of G′ and G″ at the LVR also decreased with increased storage. The magnitude of the viscoelastic moduli for G′_LVR_ was in accordance with the previously reported values for some other food hydrocolloids [27]. As for the loss-tangent values (tanδ_LVR_), which is the ratio between G′_LVR_ and G″_LVR_, all values were in the range of 0.09–0.22, indicating a predominantly elastic behavior. Moreover, the flow point (G′ = G″) indicates the stress at which the first non-linear structural change occurs. In this study, the HIPE (EΦ75%) had higher oscillatory stress values at the flow point (21–66 Pa) compared to emulsions EΦ70% (5–11 Pa) and EΦ65% (1–4 Pa), indicating that it undergoes structural changes at higher stress values.

In summary, the results of the amplitude sweep test determined the structural strength and allowed us to distinguish between weaker and stronger gels. EΦ75% was the strongest gel throughout storage as it remained in the LVE region (higher OS_L_) for a longer period of time, and it presented higher G′_LVR_ (Pa), G″_LVR_(Pa), FP_OS_ (Pa), and FP_G_ (Pa). The results also demonstrate that, with the increase in storage days, the strength of the entire system decreased. Nonetheless, it can be assumed that EΦ75% is the most stable system, as it is the strongest gel, followed by EΦ70% and EΦ65%.

##### Frequency Sweep

To further investigate the viscous and elastic behavior changes under increased frequency applications, a frequency sweep analysis at 0.1 Pa (within the LVR for all samples and days) was conducted (Figure 3c). Throughout the 60 days of storage for all samples, no crossover point (G′ = G″) was detected and G′ was always higher than G″ (data not shown). These results indicate that, within the tested experimental range (0.01–10 Hz), all samples displayed gel-like behavior—more similar to a solid rather than a liquid. Therefore, the deformations can be considered as essentially elastic and recoverable [28]. Moreover, the results suggest that, even at higher frequencies, the rheological responses of both emulsion and the HIPE have no obvious effect by the applied deformation rate. This behavior was also observed in other studies in the literature [25,28].

At a fixed frequency of 1 Hz, the tanδ values of EΦ75%, EΦ70%, and EΦ65% were within the ranges of 0.08–0.14, 0.12–0.18, and 0.16–0.22, respectively, indicating that all the samples were more elastic than viscous throughout the 60 days of storage (Table 4). In addition, it can be noted that the EΦ75% had the lowest values of tanδ, indicating that it has a stronger gel structure, which is in accordance with the apparent viscosity and amplitude sweep tests.

To further investigate the frequency dependency of G′ values, a power law relation was applied [28,29] and the degree of frequency dependence was determined by power law parameters (Table 4).

Low *n*′ values are characteristic of elastic gels, *n*′ values near to 1 indicate that the system behaves as a viscous gel, and at *n*′ values close to zero, the G′ value does not change with the frequency [28]. As can be observed in Table 4, EΦ75% had the lowest values of *n*′, presenting values very close to 0. These results confirm that EΦ75% has the least dependency on frequency changes, which can be visualized in Figure 3c, where at any given frequency G′ plot for EΦ75% barely has a slope, indicating there are minimal changes in the G′ value. The highest k′ values were also found to be from the EΦ75% sample, which means that these samples had stronger elastic structures than the others. This value also decreased with the increase in storage days for all samples (except for EΦ75%), which can be attributed to the formation of a weaker network.

##### Temperature Sweep

**Figure 4:**
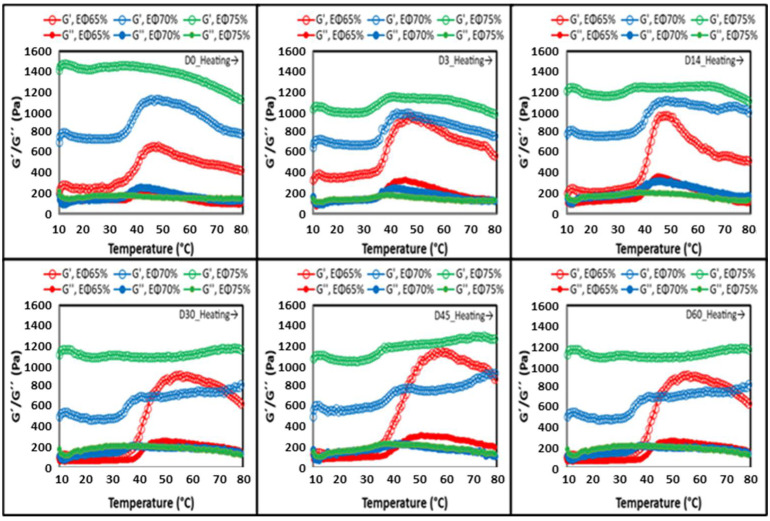
Temperature sweeps (heating) for EΦ65%, EΦ70%, and EΦ75% throughout 60 days storage at 25 °C.

To determine the sensitivity of the sample structure to thermal changes, a temperature sweep was conducted. All samples had predominance of elastic behavior over the viscous (G′ > G″) and formed structured gels with different strengths. No melting was detected as G′ did not equal G″ in the entire temperature range evaluated, indicating that all samples remained in a solid-like state. From 10 °C to around 40 °C, all samples showed a slight G′ and G″ decrease. However, at temperatures above 40 °C, an increase in G′ and G″ values was observed for both emulsions (EΦ65% and EΦ70%), behavior which was observed only slightly in the HIPE (EΦ75%) (Figure 4). This increase in G′ can be related to the development and transformation of the liquid state into a gel state (sol–gel transition) and/or due to the thickening effect of the starches leached out from the chickpea seed, which restricts the mobility of fluids. [28]. Similar results were obtained in other studies using protein, such as with whey protein emulsions [30] and emulsion gels stabilized by pea flour [31].

Within the days of storage, it was possible to observe that, for EΦ65%, already after day 0, G′ started to considerably increase, followed by a sharp decrease (Figure 4). Following day 14, G′ even became higher than EΦ70%. This behavior was not observed in EΦ70% and EΦ75%, which remained with similar behaviors in every temperature scan throughout the 60 days of storage.

The structure of the studied emulsions and HIPE was evaluated through a correlation between G′ and G″ (G′/G). At a G′/G″ ratio lower than 10, the gel is considered a weak gel; whereas, if this value is above 10, the gel is considered a strong gel [32]. At the maximum temperature of analysis (80 °C), the calculation for the gel strength of these systems was performed. The ratios were 7.5, 5.8, and 4.6, for EΦ75%, EΦ70%, and EΦ65%, respectively, on day 0, indicating that the gel strength of the samples increased at higher concentrations of oil. In summary, a higher Φ concentration increased the apparent viscosity and viscoelasticity of both emulsions and the HIPE.

### 3.4. Conventional Emulsions and HIPE Stability

**Figure 5:**
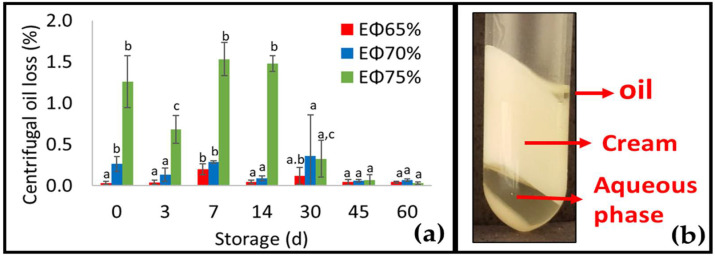
Oil loss upon centrifugation throughout 60 days of storage at 25 °C (**a**) and appearance of samples after centrifugation (**b**). Mean values (mean value ± standard derivation, *n* = 3) for the same parameter, sample, and column with different lower-case superscripts are significantly different based on Tukey’s test at *p* < 0.05.

Following centrifugation, all the samples were separated into three layers as was reported in previous studies with protein HIPEs [16,33,34]. The top layer consisted of the oil fraction and was quantified in terms of oil loss upon centrifugation (Figure 5a). The middle layer consisted of a cream layer and the bottom layer was an aqueous phase. Up to storage day 14, the oil loss was higher at the higher oil concentration (EΦ75% > EΦ70% > EΦ65%). These results may be related to structuring agent concentration, as there was probably not enough structurant available to be adsorbed on the surface of oil droplets at higher oil concentrations. Interestingly, after 14 d of storage, the oil loss was considerably reduced, indicating that a rearrangement might have occurred. Although the oil loss upon centrifugation showed a significant difference (*p* < 0.05) by the Tukey’s test, the variation between days 0 and 30 did not exceed 1.5, 0.3, and 0.2% for EΦ75%, EΦ70%, and EΦ65%, respectively, which can be considered quite low.

## 4. Conclusions

In this study, solely chickpea aquafaba at different oil phase volumes (Φ) of canola oil were used to produce stable emulsions and HIPE. The results indicate that the emulsions resist prolonged storage without breakdown through physical instability mechanisms. In terms of the rheological properties, all samples showed gel-like behavior throughout 60 days of storage at 25 °C. Moreover, the rheological tests showed a weakening of the gel’s strength throughout storage. Among the three studied formulations, EΦ75% showed to be the most stable system in terms of droplet size and rheological properties. In addition, samples had less than 1.5% of oil loss after centrifugation, which indicated that all samples had very good centrifugation stability. The outcome of this study produced an inexpensive potential substitute for saturated and trans-fat for food products, in addition to the valuable utilization of biowaste from the food industry and its conversion into a high value food ingredient. Moreover, the CA-based emulsions and HIPE did not require any modification method prior to production (i.e., sonication) nor further cumbersome processes (i.e., gelling, complexing, and heating) or the use of expensive equipment, and thus no high additional operational costs. Nonetheless, to study the feasibility and acceptability of using these systems as a fat replacer, there is a need for application in a food system, as well as consumer sensory analysis.

## Figures and Tables

**Table 1:** Droplet mean diameter (D_[4,3]_ and D_[3,2]_) of the emulsions and HIPE during 60 days of storage at 25 °C.

	D_[4,3]_ (µm)			D_[3,2]_ (µm)		
Day	EΦ65%	EΦ70%	EΦ75%	EΦ65%	EΦ70%	EΦ75%
0	14.1 ± 0.1 ^aA^	12.3 ± 0.3 ^aB^	11.0 ± 0.2 ^aC^	6.5 ± 0.1 ^aA^	5.9 ± 0.1 ^abB^	5.5 ± 0.0 ^bC^
3	15.3 ± 0.5 ^bcA^	12.5 ± 0.2 ^abB^	11.4 ± 0.1 ^abC^	7.0 ± 0.2 ^bA^	6.0 ± 0.1 ^bcB^	5.4 ± 0.0 ^abC^
7	14.4 ± 0.8 ^abA^	12.8 ± 0.6 ^abB^	11.4 ± 0.2 ^abC^	6.3 ± 0.4 ^aA^	5.9 ± 0.4 ^abB^	5.4 ± 0.1 ^abC^
30	15.9 ± 1.3 ^cA^	12.9 ± 0.6 ^bB^	11.5 ± 0.2 ^bC^	7.0 ± 0.2 ^bA^	6.2 ± 0.2 ^cB^	5.7 ± 0.1 ^bC^
45	14.6 ± 0.4 ^abA^	12.5 ± 0.4 ^abB^	11.5 ± 0.3 ^abC^	6.6 ± 0.2 ^acA^	5.7 ± 0.1 ^aB^	5.4 ± 0.0 ^aC^
60	14.8 ± 0.6 ^abA^	13.0 ± 0.3 ^bB^	12.8 ± 0.8 ^cB^	6.9 ± 0.1 ^bcA^	5.9 ± 0.1 ^abB^	5.7 ± 0.1 ^cC^

Mean values (mean value ± standard derivation, *n* = 9) for the same parameter and column with different lower-case superscript are significantly different based on Tukey’s test at *p* < 0.05. Mean values for the same parameter and row with different upper-case superscript are significantly different by the Tukey’s test at *p* < 0.05.

**Table 2:** Flow consistency index (k) and flow behavior index (*n*) of the power law model (R^2^ > 0.99), and apparent viscosity (η) at a shear rate of 5 (η_5_) and 300 s^−1^ (η_300_) and the initial shear stress (σ_0_) throughout the 60 days of storage at 25 °C.

		$\mathrm{Model}\;\mathrm{Parameters}\;(\sigma =k\times \gamma^{n})$		Experimental Parameters		
	Storage (d)	k (Pa·s^n^)	*n*	η_5_ (Pa·s)	η_300_ (Pa·s)	σ_0_ (Pa)
EΦ65%						
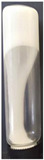	0	4.75 ± 0.15 ^ab^	0.48 ± 0.01 ^abc^	1.83 ± 0.03 ^ab^	0.25 ± 0.00 ^a^	6.54 ± 0.26 ^a^
	3	5.81 ± 0.21 ^a^	0.47 ± 0.00 ^c^	2.27 ± 0.15 ^ab^	0.28 ± 0.01 ^ab^	8.47 ± 0.68 ^a^
	7	5.84 ± 0.52 ^a^	0.47 ± 0.01 ^ac^	2.34 ± 0.27 ^b^	0.29 ± 0.02 ^b^	8.50 ± 0.96 ^a^
	14	5.19 ± 0.62 ^ab^	0.48 ± 0.01 ^abc^	2.14 ± 0.25 ^ab^	0.27 ± 0.01 ^a b^	7.86 ± 0.92 ^a^
	30	4.75 ± 0.60 ^ab^	0.49 ± 0.01 ^ab^	1.94 ± 0.22 ^b^	0.26 ± 0.01 ^ab^	7.03 ± 1.15 ^a^
	45	4.80 ± 0.24 ^ab^	0.49 ± 0.00 ^ab^	1.95 ± 0.15 ^ab^	0.27 ± 0.01 ^ab^	7.17 ± 0.73 ^a^
	60	4.40 ± 0.04 ^b^	0.50 ± 0.00 ^b^	1.80 ± 0.06 ^a^	0.26 ± 0.00 ^ab^	6.41 ± 0.28 ^a^
EΦ70%						
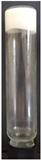	0	10.86 ± 0.65 ^a^	0.41 ± 0.00 ^ab^	4.02 ± 0.24 ^a^	0.37 ± 0.02 ^a^	16.47 ± 1.17 ^a^
	3	13.12 ± 0.26 ^cd^	0.40 ± 0.00 ^ac^	4.82 ± 0.36 ^a^	0.43 ± 0.02 ^b^	20.25 ± 1.04 ^bc^
	7	13.49 ± 0.44 ^d^	0.39 ± 0.00 ^c^	4.83 ± 0.43 ^a^	0.43 ± 0.02 ^b^	20.44 ± 1.00 ^c^
	14	11.95 ± 0.66 ^abcd^	0.40 ± 0.00 ^abc^	4.47 ± 0.39 ^a^	0.40 ± 0.01 ^ab^	18.55 ± 1.58 ^abc^
	30	10.94 ± 0.71 ^ab^	0.41 ± 0.00 ^b^	4.13 ± 0.31 ^a^	0.39 ± 0.02 ^ab^	16.65 ± 1.47 ^ab^
	45	12.63 ± 1.00 ^bcd^	0.41 ± 0.00 ^abc^	4.62 ± 0.49 ^a^	0.43 ± 0.02 ^b^	18.59 ± 2.06 ^abc^
	60	11.74 ± 0.24 ^abc^	0.41 ± 0.01 ^ab^	4.16 ± 0.21 ^a^	0.41 ± 0.01 ^ab^	16.67 ± 0.48 ^ab^
EΦ75%						
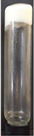	0	26.69 ± 2.93 ^a^	0.32 ± 0.01 ^abc^	8.96 ± 0.86 ^a^	0.55 ± 0.06 ^a^	40.67 ± 4.33 ^a^
	3	33.93 ± 3.09 ^ab^	0.31 ± 0.00 ^a^	11.31 ± 1.30 ^a^	0.65 ± 0.07 ^ab^	50.55 ± 5.73 ^ab^
	7	31.28 ± 3.19 ^ab^	0.31 ± 0.01 ^abc^	10.07 ± 1.20 ^a^	0.63 ± 0.03 ^ab^	46.28 ± 4.77 ^ab^
	14	35.74 ± 3.98 ^b^	0.31 ± 0.01 ^ab^	11.27 ± 1.22 ^a^	0.69 ± 0.04 ^b^	53.37 ± 5.72 ^b^
	30	28.54 ± 1.10 ^ab^	0.32 ± 0.01 ^abc^	9.42 ± 0.23 ^a^	0.61 ± 0.04 ^ab^	42.90 ± 1.20 ^ab^
	45	31.02 ± 1.92 ^ab^	0.32 ± 0.00 ^bc^	10.37 ± 0.61 ^a^	0.66 ± 0.02 ^ab^	44.55 ± 2.67 ^ab^
	60	30.45 ± 1.41 ^ab^	0.33 ± 0.01 ^c^	9.88 ± 0.51 ^a^	0.66 ± 0.01 ^ab^	42.45 ± 2.22 ^ab^

σ is shear stress (Pa); k is flow consistency index (Pa·s)^n^; γ is shear rate (1/s); and *n* is flow behavior index (dimensionless). Mean values (mean value ± standard derivation, *n* = 3) for the same parameter, sample, and column with different lower-case superscripts are significantly different based on Tukey’s test at *p* < 0.05.

**Table 3:** Rheological parameters of storage (G′_LVR_) and loss (G″_LVR_) moduli at the linear viscoelastic region (LVR), limiting value of oscillatory stress (OS_L_), the loss-tangent (tanδ_LVR_) at the LVR, flow-point oscillatory stress (FP_OS_), and flow-point G (FP_G_) as determined by stress sweep tests (at a 1 Hz frequency) for samples stored at 25 °C.

Sample	Storage (d)	G′_LVR_ (Pa)	G″_LVR_(Pa)	OS_L_(Pa)	tanδ_LVR_	FP_OS_(Pa)	FP_G_(Pa)
	0	380.94 ± 37.04 ^ac^	58.70 ± 5.42 ^ab^	1.26 ± 0.00 ^a^	0.15 ± 0.00 ^a^	2.64 ± 0.34 ^cd^	105.27 ± 34.63 ^a^
	3	426.55 ± 43.31 ^a^	72.01 ± 7.90 ^bc^	1.26 ± 0.00 ^a^	0.17 ± 0.02 ^a^	4.03 ± 0.65 ^e^	74.56 ± 21.71 ^a^
	7	452.70 ± 8.89 ^a^	72.63 ± 1.66 ^bc^	1.26 ± 0.00 ^a^	0.16 ± 0.00 ^a^	2.84 ± 0.00 ^d^	107.11 ± 28.33 ^a^
EΦ65%	14	415.05 ± 50.79 ^ac^	81.13 ± 7.37 ^c^	1.00 ± 0.00 ^e^	0.20 ± 0.02 ^ab^	2.45 ± 0.34 ^bcd^	115.47 ± 26.41 ^a^
	30	318.57 ± 47.74 ^bc^	59.10 ± 10.2 ^ab^	0.74 ± 0.09 ^d^	0.19 ± 0.02 ^ab^	1.61 ± 0.26 ^ab^	79.37 ± 35.80 ^a^
	45	243.02 ± 34.13 ^b^	53.62 ± 1.02 ^a^	0.63 ± 0.00 ^c^	0.22 ± 0.03 ^b^	1.32 ± 0.17 ^a^	81.25 ± 8.28 ^a^
	60	215.58 ± 30.05 ^b^	47.46 ± 5.58 ^a^	0.50 ± 0.00 ^b^	0.22 ± 0.01 ^b^	1.79 ± 0.00 ^abc^	58.21 ± 5.13 ^a^
	0	695.53 ± 39.37 ^a^	76.11 ± 3.66 ^b^	5.01 ± 0.00 ^e^	0.11 ± 0.00 ^a^	10.52 ± 1.34 ^bc^	181.60 ± 17.44 ^ab^
	3	742.63 ± 27.58 ^a^	84.90 ± 2.52 ^ab^	5.01 ± 0.00 ^e^	0.11 ± 0.00 ^ab^	12.27 ± 1.69 ^c^	147.08 ± 7.24 ^a^
	7	753.74 ± 50.41 ^a^	97.10 ± 12.31 ^a^	3.98 ± 0.00 ^d^	0.13 ± 0.01 ^bc^	8.36 ± 1.07 ^abc^	203.23 ± 12.49 ^ab^
EΦ70%	14	764.28 ± 42.70 ^a^	99.46 ± 4.70 ^a^	3.71 ± 0.47 ^cd^	0.13 ± 0.00 ^cd^	6.56 ± 2.74 ^ab^	227.96 ± 34.67 ^b^
	30	749.47 ± 26.23 ^a^	91.81 ± 3.21 ^ab^	3.16 ± 0.00 ^bc^	0.12 ± 0.00 ^abc^	7.13 ± 0.00 ^ab^	241.78 ± 43.84 ^b^
	45	661.30 ± 38.73 ^a^	95.45 ± 5.11 ^a^	2.73 ± 0.38 ^ab^	0.14 ± 0.00 ^d^	5.49 ± 0.92 ^a^	205.81 ± 29.12 ^ab^
	60	532.30 ± 38.38 ^b^	89.48 ± 4.75 ^ab^	2.03 ± 0.46 ^a^	0.17 ± 0.01 ^e^	5.27 ± 0.67 ^a^	144.58 ± 13.76 ^a^
	0	1377.74 ± 69.46 ^c^	120.09 ± 25.22 ^a^	19.95 ± 0.00 ^d^	0.10 ± 0.01 ^a^	65.56 ± 27.44 ^b^	318.96 ± 39.47 ^a^
	3	1142.70 ± 21.02 ^a^	114.06 ± 2.20 ^a^	15.85 ± 0.00 ^c^	0.10 ± 0.00 ^a^	41.88 ± 5.34 ^ab^	241.61 ± 44.11 ^ab^
	7	1087.21 ± 27.81 ^a^	103.38 ± 6.76 ^a^	12.59 ± 0.00 ^b^	0.09 ± 0.01 ^a^	34.35 ± 2.36 ^a^	257.43 ± 17.40 ^ab^
EΦ75%	14	1190.30 ± 71.31 ^a^	117.95 ± 10.63 ^a^	12.59 ± 0.00 ^b^	0.10 ± 0.00 ^a^	35.72 ± 0.00 ^ab^	279.65 ± 54.68 ^ab^
	30	1073.52 ± 37.48 ^a^	101.29 ± 4.07 ^a^	7.94 ± 0.00 ^a^	0.09 ± 0.00 ^a^	41.88 ± 5.34 ^ab^	170.20 ± 13.41 ^b^
	45	1157.35 ± 68.12 ^a^	114.39 ± 1.69 ^a^	7.94 ± 0.00 ^a^	0.10 ± 0.00 ^a^	25.45 ± 2.92 ^a^	293.23 ± 61.55 ^a^
	60	928.37 ± 41.38 ^b^	105.39 ± 6.47 ^a^	7.40 ± 0.94 ^a^	0.11 ± 0.01 ^a^	20.99 ± 2.68 ^a^	247.10 ± 44.41 ^ab^

Flow point (G′ = G″). Mean values (mean value ± standard derivation, *n* = 3) for the same parameter, sample, and column with different lower-case superscripts are significantly different based on Tukey’s test at *p* < 0.05.

**Table 4:** Power law parameters (k′ and *n*′) for the storage modulus G′ throughout the 60 days of storage at 25 °C and the storage (G′) and loss (G″_LVR_) moduli; the loss-tangent (tanδ) at a frequency of 1 Hz for samples stored at 25 °C.

		$\mathrm{Model}\;\mathrm{Parameters}\;(G^{\prime }=k^{\prime }\times \omega^{n^{\prime }})$			Experimental Parameters		
Samples	Storage (d)	k′ (Pa·s^n^)	*n*′	R^2^	G′ (Pa)	G″ (Pa)	tanδ
	0	394 ± 32 ^ab^	0.18 ± 0.02 ^a^	0.96	599 ± 54 ^a^	109 ± 12 ^abc^	0.18 ± 0.01 ^a^
	3	461 ± 4 ^abc^	0.22 ± 0.05 ^a^	0.97	718 ± 11 ^a^	134 ± 8 ^c^	0.19 ± 0.01 ^a^
	7	489 ± 27 ^c^	0.18 ± 0.03 ^a^	0.96	723 ± 53 ^a^	119 ± 6 ^bc^	0.16 ± 0.00 ^a^
EΦ65%	14	487 ± 30 ^bc^	0.19 ± 0.02 ^a^	0.97	654 ± 89 ^a^	130 ± 13 ^c^	0.20 ± 0.04 ^a^
	30	367 ± 46 ^a^	0.17 ± 0.01 ^a^	0.96	546 ± 55 ^ab^	96 ± 11 ^ab^	0.18 ± 0.02 ^a^
	45	273 ± 36 ^d^	0.19 ± 0.02 ^a^	0.96	425 ± 36 ^b^	92 ± 14 ^ab^	0.22 ± 0.05 ^a^
	60	266 ± 31 ^d^	0.19 ± 0.02 ^a^	0.96	408 ± 49 ^b^	79 ± 13 ^a^	0.19 ± 0.03 ^a^
	0	846 ± 58 ^ab^	0.14 ± 0.02 ^ab^	0.98	1097 ± 42 ^a^	172 ± 12 ^a^	0.16 ± 0.01 ^ab^
	3	914 ± 20 ^b^	0.17 ± 0.01 ^b^	0.99	1396 ± 163 ^c^	250 ± 40 ^b^	0.18 ± 0.03 ^b^
	7	810 ± 56 ^ab^	0.13 ± 0.02 ^ab^	0.95	1069 ± 37 ^ab^	145 ± 27 ^a^	0.14 ± 0.02 ^ab^
EΦ70%	14	828 ± 53 ^ab^	0.14 ± 0.02 ^ab^	0.97	1105 ± 109 ^a^	174 ± 36 ^a^	0.16 ± 0.02 ^ab^
	30	731 ± 40 ^ac^	0.12 ± 0.00 ^a^	0.96	972 ± 43 ^ab^	115 ± 6 ^a^	0.12 ± 0.01 a ^a^
	45	709 ± 71 ^ac^	0.14 ± 0.01 ^ab^	0.97	968 ± 79 ^ab^	151 ± 18 ^a^	0.16 ± 0.01 ^ab^
	60	617 ± 22 ^c^	0.15 ± 0.01 ^ab^	0.97	845 ± 38 ^b^	130 ± 8 ^a^	0.15 ± 0.00 ^ab^
	0	1252 ± 97 ^a^	0.07 ± 0.01 ^a^	0.97	1357 ± 51 ^a^	120 ± 29 ^a^	0.09 ± 0.02 ^ab^
	3	1244 ± 18 ^a^	0.08 ± 0.00 ^a^	0.91	1447 ± 17 ^ab^	122 ± 5 ^a^	0.08 ± 0.00 ^a^
	7	1207 ± 98 ^a^	0.09 ± 0.02 ^ab^	0.96	1419 ± 80 ^ab^	165 ± 36 ^ab^	0.12 ± 0.03 ^abc^
EΦ75%	14	1326 ± 43 ^a^	0.09 ± 0.00 ^ab^	0.96	1502 ± 162 ^ab^	182 ± 46 ^ab^	0.12 ± 0.02 ^abc^
	30	1128 ± 82 ^a^	0.08 ± 0.01 ^a^	0.97	1342 ± 87 ^a^	124 ± 19 ^a^	0.09 ± 0.01 ^abc^
	45	1183 ± 26 ^a^	0.11 ± 0.01 ^b^	0.98	1698 ± 224 ^b^	226 ± 19 ^b^	0.13 ± 0.01 ^bc^
	60	1122 ± 76 ^a^	0.11 ± 0.01 ^b^	0.96	1375 ± 89 ^ab^	187 ± 28 ^ab^	0.14 ± 0.01 ^c^

Mean values (mean value ± standard derivation, *n* = 3) for the same parameter, sample, and column with different lower-case superscripts are significantly different based on Tukey’s test at *p* < 0.05.

## Data Availability

The data used in this study are available in this article.
